# Thermal melt circular dichroism spectroscopic studies for identifying stabilising amphipathic molecules for the voltage‐gated sodium channel NavMs

**DOI:** 10.1002/bip.23067

**Published:** 2017-09-19

**Authors:** Sam M. Ireland, Altin Sula, B.A. Wallace

**Affiliations:** ^1^ Institute of Structural and Molecular Biology, Birkbeck College, University of London London United Kingdom

**Keywords:** amphipols, detergents, circular dichroism spectroscopy, membrane proteins, thermal stability

## Abstract

Purified integral membrane proteins require amphipathic molecules to maintain their solubility in aqueous solutions. These complexes, in turn, are used in studies to characterise the protein structures by a variety of biophysical and structural techniques, including spectroscopy, crystallography, and cryo‐electron microscopy. Typically the amphilphiles used have been detergent molecules, but more recently they have included amphipols, which are polymers of different sizes and compositions designed to create smaller, more well‐defined solubilised forms of the membrane proteins. In this study we used circular dichroism spectroscopy to compare the secondary structures and thermal stabilities of the NavMs voltage‐gated sodium channel in different amphipols and detergents as a means of identifying amphipathic environments that maximally maintain the protein structure whilst providing a stabilising environment. These types of characterisations also have potential as means of screening for sample types that may be more suitable for crystallisation and/or cryo‐electron microscopy structure determinations.

## 1 INTRODUCTION

Membrane proteins require amphipathic molecules for solubility and stability in aqueous solutions.^1^ In many cases the amphipathic molecules of choice are detergents. Indeed, most crystal structures of membrane proteins to date have been of detergent complexes. However, more recently amphipols, which are amphipathic polymers,^2^ have been shown to be effective alternative environments in which membrane proteins can retain their structure and function. They have proven to be of particular value in producing samples for high resolution cryo‐electron microscopic (cryo‐EM) studies, now an increasingly important method for three‐dimensional structure determination.^3^ There has also been one example of lipid mesophase crystals created by the transfer of an amphipol‐solubilised membrane protein.^4^ Considerations in the choice of amphipathic molecules are the ability to maintain the structural and functional integrity of the protein in the amphipathic complex, as well as to match the sizes and shapes of the hydrophobic and hydrophilic regions of the protein to the amphiphile structures.

Circular dichroism (CD) spectroscopy is an important method for characterising membrane protein structure and stability.^5^ It can provide information not only on the secondary structure of proteins in amphiphiles, but also on the thermal stability of membrane proteins in these environments. To date, most such studies have concentrated on detergent complexes, but the increasing use of amphipol complexes in structural studies has suggested that comparisons of protein complexes in both detergents and amphipols would be valuable for assessing their suitability as membrane‐mimetic environments.

We have chosen NavMs (the prokaryotic voltage‐gated sodium channel from *M. marinus*), as the exemplar protein for these studies. Its crystal structure has been determined at high resolution^6^ [giving us a comparison for the secondary structures calculated from CD spectroscopy] and it can be isolated in, and/or transferred to, a number of different amphipathic environments. NavMs was also chosen because it has a particularly simple secondary structure: it is highly helical protein, with some unstructured regions, but has no apparent beta‐sheet content. Hence it provides a system that enables us to monitor the specific unfolding/refolding of helical secondary structural elements.

## 2 MATERIALS AND METHODS

### 2.1 Materials

The detergents dodecyl maltoside (DDM), 5‐cyclohexyl‐1‐pentyl‐β‐d‐maltoside (Cymal5), and decanoyl‐*N*‐hydroxyethylglucamide (HEGA10), and the amphipols A835 [average MW ∼8 kDa] and poly(maleic anhydride‐alt‐1‐decene) substituted with 3‐(dimethylamino)propylamine (Pmal‐C8) [average MW ∼18.5 kDa] were all obtained from Anatrace.

### 2.2 Purification and preparation of NavMs/amphiphile complexes

The full‐length NavMs protein (Uniprot ID A0L5S6) was purified according to the method previously described,^6^ following solubilisation in 1.5% DDM. The protein was loaded onto a 1 mL His‐trap column (GE Healthcare). Bound protein was eluted with 0.1% DDM, 0.52% HEGA10, or 0.3% Cymal5. It was then purified by size exclusion chromatography (SEC) using a Superdex 200 10/300 column (GE Healthcare) in the cognate detergents. The His‐tag was removed by thrombin (Novagen) cleavage, followed by a second SEC step again in the appropriate detergent at a concentration above its critical micelle concentration, as previously described.^6^ Samples were concentrated using a 100 kDa cut‐off Amicon concentrator. The corresponding flow‐throughs from the concentration step (which did not contain any protein) were used as the spectroscopic baselines for each type of sample.

For the amphipol samples, 0.5 mg of purified protein in 0.52% HEGA10 (from above) was mixed with 2.5 mg of either A835 or Pmal‐C8 and incubated overnight. Following SEC, protein peak fractions were pooled and concentrated as described above.

Protein concentrations were determined from the A280 value measured in a Nanodrop spectrophotometer, using a calculated extinction coefficient of 29450 M^−1^ cm^−1^.^7^ The absence of detectable amounts of the starting HEGA10 detergent (which absorbs in the far UV region) in the amphipol samples was evident from the far UV spectra.

### 2.3 CD spectroscopy

In each case, the sample contained purified protein at ∼1.2 mg mL^−1^ and both the sample and baseline contained 20 mM Tris, pH 7.5, 100 mM NaF, and 8% glycerol. NaF was used instead of NaCl to enable data collection to lower wavelengths.^8^ All data was collected on an Aviv 430 circular dichroism instrument, using the same quartz Suprasil (Hellma Ltd.) “bottle” cell with a pathlength of 0.0093 cm.

In general for each sample type, two independent sample preparations were made, and for each of the preparations, one or two spectral runs were undertaken. For each spectral run, three repeat spectra were collected at each temperature over the wavelength range from 280 to 190 nm in 1‐nm steps, and over the temperature range from 20°C to at least 80°C in 5°C increments, with measurements at each temperature following a 3‐min equilibration period. The first and third spectra measured at each temperature were compared to establish that the sample had come to equilibrium at that temperature prior to the data being collected. For each sample type, at least one spectral run was cooled to 20°C after heating to 80°C, after which three repeat spectra were obtained to enable comparison with the initial 20°C spectrum for that sample.

For each type of amphipathic sample preparation, three repeat baseline spectra were also obtained at each temperature. These consisted of the flow‐through from the exchange/concentration step in the cognate detergent or amphipol.

### 2.4 Data processing

Data processing was carried out using CDTool software^9^ as follows: Replicate scans of each sample were scaled, averaged and the corresponding baselines were subtracted from the averaged sample spectra. The spectra were smoothed with a Savitsky–Golay filter, zeroed between 263 and 270 nm, and converted to delta epsilon values using a mean residue weight of 112.9 kDa.

### 2.5 Secondary structure analyses

Secondary structures were calculated using the DichroWeb analysis server^10^ with the CONTIN algorithm^11^, ^12^ and the SMP180 reference dataset designed for use with membrane proteins.^13^ The NRMSD parameter calculated is a measure of the goodness‐of‐fit of the calculated secondary structure to the experimental data.^10^ For all examples described in this study the NRMSD values were <0.1, indicating strong correspondence between the data and the calculated secondary structures.

The secondary structure of NavMs derived from its crystal structure in HEGA10 detergent [PDBID 5HVX] was determined using the DSSP algorithm.^14^

### 2.6 Thermal melt analyses

Single wavelength melt curves were obtained by plotting the measured values of the 223 and 194 nm peaks of each spectrum against temperature. Curves were normalized by setting the peak value at 20°C to 1.0 for each sample type and scaling the other spectra from the series with the same normalisation factor.

The principal component analyses (PCA) for each sample type were produced using CDTool software.^9^ The two main components in the PCA analyses corresponded to spectra of folded and unfolded structures, respectively, and their magnitudes were plotted as a function of temperature, providing an overall indication of the thermal stability of the protein.

Indirect *T*_m_ determinations for each sample type plotted the calculated helical secondary structures at each temperature point versus the temperature.

## 3 RESULTS AND DISCUSSION

### 3.1 Choice of samples

NavMs or its pore‐only derivative have previously been isolated and purified in a number of detergents (decylmaltoside, nonyl maltoside, dodecylmaltoside, Cymal5, and HEGA10) 6, 15 and crystals have been produced from both HEGA10 and nonyl maltoside detergents.^6^, ^16^ However, the HEGA10 detergent is not suitable for CD experiments as it includes an glucamide chromophore, which due to its absorbance and dichroism, precludes measurements at wavelengths below ∼225 nm. Cymal5 and dodecyl maltoside (chosen as exemplars of the maltoside headgroup family of detergents, but with different types of hydrophobic chains) are suitable for optical measurements in the far UV wavelength range. They were used to compare the effects of detergent molecules of different sizes and with different critical micelle concentrations.

Whilst detergents are generally used for crystallography (and indeed NavMs crystals diffracted to high resolution (2.45 Å) in HEGA10), amphipols have been shown to be more suitable for cryoEM structure determinations of a number channel of proteins.^17^ Hence, in this study comparisons were made not only between detergents and amphipols, but also between two different types of amphipols and two different types of detergents.

### 3.2 Comparison of secondary structures in different environments

**Figure 1:**
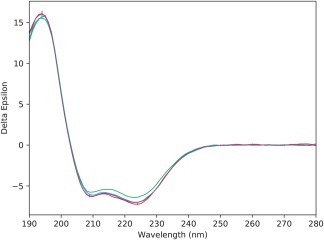
Circular dichroism spectra of NavMs in different amphiphiles. CD spectra of NavMs in Pmal‐C8 (red), A835 (blue), DDM (green), and Cymal5 (purple). The error bars correspond to 1 s.d. in repeated measurements

The spectra (Figure 1) and the secondary structures calculated from the spectra (Table 1) obtained for samples at 20°C in all environments were virtually identical, suggesting the process of solubilisation did not in itself have a significant effect on the protein structure. All produced calculated helical secondary structures of ∼68% (Table 1), which correspond closely to the α‐helix content (67%) seen in the crystal structure.

**Table 1:** Calculated secondary structure content (% helix) of NavMs in different environments at 20° and 80°C

Amphiphile	20°C	80°C
Pmal‐C8	68	52
A835	68	30
DDM	68	28
Cymal5	69	22

### 3.3 Monitoring unfolding as a function of temperature

**Figure 2:**
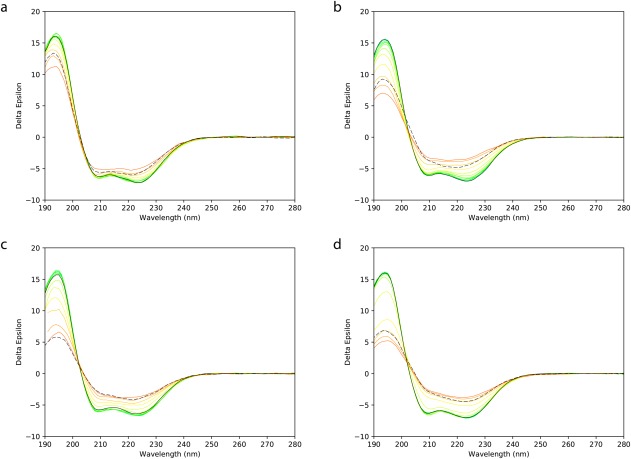
Thermal melt plots as a function of temperature. Overlaid CD spectra obtained at different temperatures ranging from 20°C to 80°C. In each case the bottom line (green) is for the spectrum obtained at 20°C; the series of rainbow‐coloured lines are plots obtained at successive 5°C intervals, and the spectrum obtained after cooling to 20°C is depicted by a black dotted line. These are for samples in (a) Pmal‐C8, (b) A835, (c) DDM, and (d) Cymal5

The different rates of change in the spectra as a function of temperature in the different environments were visually evident from the melt series spectra (Figure 2A‐D). The peak at ∼223 nm is almost entirely the result of helical secondary structures. The peak at 194 nm will have a maximal value for helical structures, but be decreased in magnitude and shifted to higher wavelengths, as seen in the thermal melts (Figure 2), when disordered (unfolded) structure is present. However, it will also be depressed in magnitude if protein aggregation occurs.

**Figure 3:**
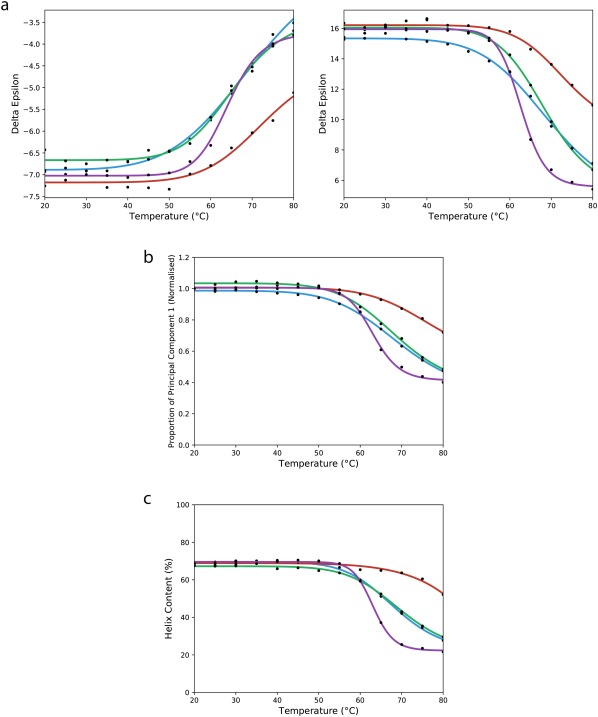
Changes in spectral features as a function of temperature. (a) The θ_223_ (left) and θ_194_ (right) values are plotted as a function of temperature for all four amphiphiles, and the curves fit to a sigmoidal function. The same colour‐coding is used in all three parts of this figure: Pmal‐C8 is in red, A835 is in blue, DDM is in green, and Cymal5 is in purple. (b) Principal component analyses as a function of temperature. The proportion of component 1 (folded) is plotted as a function of temperature for all four sample types. (c) Calculated α–helical secondary structures as a function of temperature

In the Pmal‐C8 series, both the peak magnitudes at 223 and 194 nm (Figure 3A) and the calculated helical content of the protein (Table 1) decreased by ∼25% at 80°C when compared with the values at 20°C. In contrast, in DDM and the A835 amphipol, the spectral magnitudes of the 223 nm and 194 nm peaks decreased by ∼50% (Figure 3A) at 80°C compared to the 20°C peaks, whereas for Cymal5, although the decrease of the 223 nm peak was comparable, the 194 nm peak decrease was more substantial (decreasing by ca. 70%), suggesting that the protein not only unfolded more, but likely formed aggregates in this detergent. In both detergents and the A835 amphipol, the corresponding decrease in the calculated helical secondary structure was much more substantial than for Pmal‐C8 (Table 1) at 80°C. All of these results suggest the protein was most stable in Pmal‐C8. It is, however, notable that even at the highest temperatures (Table 1) the protein was not completely unfolded in any of the amphiphiles, consistent with previous observations for other prokaryotic and eukaryotic sodium channels^18^, ^19^, ^20^ which showed a significant amount of helical structure present even at high temperatures.

### 3.4 Estimation of relative T_m_ values

*T*_m_ values (the temperature point where 50% of the total change occurs) can often be estimated from thermal unfolding data in two different direct ways and one indirect way. The direct ways include: (1) plotting the value of the change that occurs at a single wavelength as a function of temperature [this can be done for several peaks to determine if the changes are concerted throughout the molecular features], and (2) using PCA and plotting the changes in the fractions of native and unfolded the principal components as a function of temperature. The latter method effectively uses the values at all wavelengths of the spectrum simultaneously, providing an overall comparison of the relative stabilities of the whole protein in the different environments, as opposed to single features. The indirect method for estimating *T*_m_ utilises the secondary (derived) data (in this case, helix content), rather than a directly measured parameter, at each temperature and plots that versus temperature.

In this study, however, the NavMs protein was observed to be remarkably stable at all temperatures and hence did not achieve a fully denatured state even at 80°C; consequently it has not been possible to accurately calculate the *T*_m_ since the curves did not achieve plateaux at the highest temperatures. However, as noted above, the single wavelength curves (Figure 3A) both suggested that the maximal extent of unfolding as a function of temperature was also the smallest for Pmal‐C8, and that the gradient of unfolding changes as a function of temperature was also less for the Pmal‐C8 sample. From the principal component analysis curves (Figure 3B), it is also clear that the total amount of component 1 (folded) lost at 80°C differs in all three cases, with the Pmal‐C8 sample again retaining the most native structure (∼0.7 of component 1), the Cymal5 sample retaining the least (∼0.4) and the DDM and A835 samples retaining roughly the same amount (∼0.5) of native structure at the highest temperature. Furthermore the estimated midpoints of the curves (the temperature where the component 1 value equals one‐half the component 1 value at 80°C) also differed significantly: ∼70°C for Pmal‐C8, ∼62°C for Cymal5, and ∼67°C for the other two. Together these PCA analyses suggest an order of stability of: Pmal‐C8 ≫ A835∼DDM > Cymal5. The PCA analyses can provide another hint about the process of unfolding: the relative extents of changes to the native (component 1) and unfolded (component 2) can be plotted versus temperature. As the native and unfolded component PCA curves intersect at a temperature which is the approximate midpoint of the total change (data not shown), this consistent with there being no long‐lived intermediates (in which case a third significant component (above the reproducibility levels) and a non‐sigmoidal curve would result).

The indirect analyses of thermal unfolding calculations consider the change in helical contents as a function of temperature (Figure 3C). All samples started with helical contents of ∼68% at 20°C (Table 1). For Cymal5, the final calculated helical content is ∼22% at 80°C, with the midpoint of the melt curve being at ∼63°C, whereas for Pmal‐C8, the final calculated helical content is ∼52%, with DDM and A835 having similar values of 28% and 30%, respectively. This type of analysis again suggests that Pmal‐C8 provides a more stabilising environment than the other amphiphiles.

Although all these methods for estimating T_*m*_ report on different spectral features/parameters and are not quantitatively definitive because the unfolding process is not complete for any of the samples, it is clear that the samples in Pmal‐C8 are the most thermally stable.

### 3.5 Refolding attempts

**Figure 4:**
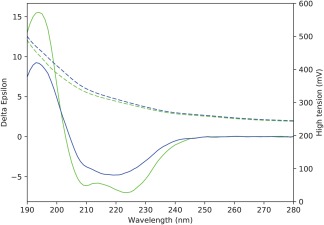
The thermal unfolding is irreversible. Comparison of the initial spectrum for the sample in A835 obtained at 20°C (green) and the spectrum obtained at 20°C after heating the sample to 80°C and then returning the temperature to 20°C (blue). The HT spectra are shown as dashed lines in the same colour as the cognate CD spectra

In none of the cases examined did the CD spectrum of the sample that was cooled to 20°C after heating to 80°C have an appearance similar to that of the initial (unheated) 20°C sample (Figures 2 and 4), indicating the samples did not refold on cooling (i.e., the process was not reversible). This is the most commonly seen result for membrane proteins^5^, ^21^, ^22^ and means that the thermodynamic parameter Δ*G* for unfolding cannot be calculated for the process.^23^ The spectra of all of the samples cooled to 20°C [Figure 2 (black dashed lines)] were also not simply a smaller version with the same shape as the spectrum of the initial 20°C sample (which would have indicated loss of material but refolding). An expanded view in Figure 4 shows, for example, that there is a significant change in shape, with the peak minimum around 223 nm blue‐shifted and the peak ∼194 nm depressed relative to the other peaks. Combined with an increased (rather than decreased) HT spectrum (which roughly corresponds to absorbance of the protein), this suggests that the decreased CD signal is not due to loss of material, but rather due to protein aggregation (Figure 4) in the cooled sample. All of the other samples showed the same trends. These results are all suggestive that the cooled to 20°C samples tended to both unfold and form irreversible aggregates upon cooling.

## 4 CONCLUSIONS

In this study, circular dichroism spectroscopy was utilised to monitor the thermal stability of a membrane protein. This method is a complementary to many other methods that are used to probe membrane stability such as fluorophore assays for unfolding,^24^ fluorescence‐detected size exclusion chromatography,^25^ blue native PAGE,^26^ and differential scanning calorimetry.^27^ Because CD measurements do not require the presence of any probe molecules (for example, either bound dyes or protein constructs including green fluorescent proteins), but do themselves provide additional structural information on the protein (secondary structural features that change), they can be a valuable addition to the cadre of methodologies for probing and characterising membrane protein stability.

This study examined the relative structures and thermal behaviours of the NavMs membrane protein in four different amphipathic environments (two detergents and two amphipols), with the aim of identifying the complex in which the protein was both most stable. At low temperatures (up to ∼40°C) in all of the amphiphiles, the protein exhibited similar circular dichroism spectra with calculated secondary structures very similar to those found in the protein crystal structure. However, in order to distinguish whether the protein was more structurally stable in any of these environments, it was then subjected to thermal stress. Not only was the native structure retained at a higher temperature in one of the amphipols, Pmal‐C8, but its unfolding profile in those samples suggested that even at higher temperatures, its secondary structure was very much intact. Comparing the two detergents, DDM appears to be more stabilising than Cymal5, which may be a consequence of its longer and less bulky hydrophobic tail, despite both detergents having the same hydrophilic head group.

In summary, this study compared the relative stabilities of the membrane protein NavMs in both detergent and amphipol environments using circular dichroism spectroscopy. These results suggest that Pmal‐C8 may be a particularly good stabilising environment for this protein and may in the future be a suitable environment for cryo‐electron microscopy, crystallography, or other biophysical characterisation studies, including drug binding. These types of studies will be particularly useful in cases such as NavMs in which the crystal structure has been determined, but crystal packing constraints prevent binding of toxins or drugs, and large conformational changes (such as those associated with gating) cannot be accommodated within the crystal lattice.

BAW dedicates this article to the memory of Mr. Jack Aviv, a major influence in the development and design of CD instruments, an advisor and friend, and an overall “Mensch.”

## ORCID

*B.A. Wallace* http://orcid.org/0000-0001-9649-5092
